# Cover

**DOI:** 10.4269/ajtmh.1104_scover

**Published:** 2024-04-02

## Abstract

Kente weaving is mesmerising artistry with profound meanings hidden within its colors and intricate symbols. It is woven on a horizontal loom to yield narrow strips, each with their own pattern, repetition, and colors. Several of these are then carefully laid out and stitched together lengthwise to make a large piece.

Evidence-based practice is akin to this process by delicately weaving intricate threads of knowledge, acquired over time from several hypotheses and observations, and eventually reveal a meaningful pattern, a fabric of understanding, as complex and finely textured as the most intricate of tapestries. *Photo Credit*: Alan Tobey/Canva

